# Prevalence of hepatitis B virus and associated risk factors among adults patients at Dessie referral and Kemise general hospitals in northeastern Ethiopia

**DOI:** 10.1002/hsr2.659

**Published:** 2022-05-22

**Authors:** Hussein Mohammed, Aragaw Eshetie, Dessie Melese

**Affiliations:** ^1^ Department of Statistics, College of Natural and Computational Science Samara University Samara Ethiopia; ^2^ Department of Statistics, College of Natural and Computational Science University of Gondar Gondar Ethiopia

**Keywords:** associated risk factors, hepatitis B virus, northeastern Ethiopia, prevalence

## Abstract

**Background and Aims:**

Hepatitis is an inflammation of the liver that can reason a variety of health problems and can be fatal. According to the most recent estimates of the Global Burden of Disease study and WHO, viral hepatitis is accountable for around 1.34 million deaths yearly, which is comparable to the yearly number of deaths from HIV/AIDS (1.3 million), malaria (0.9 million), and tuberculosis (1.3 million). This study aimed to assess the prevalence of the Hepatitis B virus and associated risk factors among adults patients at Dessie Referral and Kemise General Hospitals.

**Methods:**

The source for the data on Hepatitis B virus (HBV) was all adults aged≥18 years that were admitted and tested for HBV from September 2020 to February 2021 were included in the study. A total of 1283 adults were admitted out of which, 1080 adults have completed measurements and had been taken into consideration for this examination, and others had been excluded from the examination because of exclusion criteria. To meet our objective descriptive statistics, the *χ* ^2^ test and multiple logistic regression statistical models were used for data analysis.

**Results:**

In this study, a total of 1080 adults were included out of which 631 (58.4%) female and 449 (41.6%) were male with a mean age of 34(SD ±± 12.56) years. The overall prevalence of HBV among adults was 27.4% (95% confidence interval [CI];24.8–30.2). The results of this study showed that age 25–34(odds ratio [OR] = 3.6, *p*‐value = 0.005), 35–44 (OR = 6.67, *p*‐value <0.001), ≥45 (OR = 3.85, *p*‐value = 0.005), male (OR = 4.36, *p*‐value < 0.001), history of hospitalization (OR = 0.644, *p*‐value = 0.04), family history of HBV (OR = 1.96, *p*‐value = 0.005), and jaundice (OR = 2.50, *p*‐value = 0.005) were significant risk factors of HBV.

**Conclusion:**

The prevalence of HBV in this study is 27.4%. The results of this study showed that age, male, history of hospitalization, family history of HBV, and jaundice were significant risk factors for Hepatitis B virus.

## 1 BACKGROUND

Hepatitis B virus (HBV) infection is a major global health problem, with an estimated 290 million infections worldwide; international targets set the challenge for this public health threat to be eliminated by 2030.^1^ International Sustainable Development Goals for the eradication of HBV pollution set determined targets for 2030. There is an estimated global burden of 290 million cases of HBV infection, the majority of which are undiagnosed and untreated.^2^

According to the greatest current approximations of the Global Burden of Disease study and WHO, viral hepatitis is accountable for around 1.34 million deaths yearly, which is related to the yearly number of deaths from HIV/AIDS (1.3 million), malaria (0.9 million), and tuberculosis (1.3 million), Mortality due to viral hepatitis has increased by 63% since 1990 and is now ranked the seventh most important source of death international; however, global appreciation of the severity of the problem has not been achieved, and a global promise to combat the illness is still. In 2017, HBV infection is a common cause of viral hepatitis and affects more than 257 million people worldwide. Nearly 8% of this international load is in Sub‐Saharan Africa, with over 80,000 new infections happening every year.^3^

Additional half of the countries in the Americas (57%) have national policies for deterrence, action, and regulation of viral hepatitis. Only 54% of countries have goals for the elimination of hepatitis B.^4^

Africa is the continent with the second largest number of individuals with chronic HBV infection, with an estimated 6.1% of the adult population infected.^3^

Sub‐Saharan Africa has one of the highest burdens of disease with over 60 million living with HBV. The regional prevalence of HBV infection is about 6.1%, with approximately one in every 15 people (1:15) infected.^5^

According to various community‐based studies conducted in Ethiopia, the prevalence of HBs Ag ranges from 5.4% to 12.7%.^6^

An estimated 95% of individuals with chronic HBV infection are unaware of their infection and so do not benefit from clinical care, treatment, and interventions that are designed to reduce onward transmission.^7^

It was estimated that over 5 million people are living with chronic HBV infection among the general population of Ethiopia.^8^

Additionally, according to the WHO report, Ethiopia is observed as a country with no countrywide plan for investigation, deterrence, and control of viral hepatitis, but the country is categorized under the geographical regions with intermediate to hyperendemic viral hepatitis infections.^9^

The magnitude of the problem is not yet addressed in many parts of Ethiopia that consist of the Eastern Region. The prevalence of HBV infection varies from country to country and even from one region to another, depending on environmental factors and host characteristics. Therefore, this study tries to investigate the socio‐demographic, behavioral, and risk factors of HBV among adult patients in Northeastern Ethiopia. In line with the above reality, these studies attempted to come up with possible solutions and conclusions after having a clear understanding of the situation with the aid of using giving due emphasis to answer the subsequent studies' questions.

## 2 METHODS

### 2.1 Data source and mode of collection

Two major Hospitals that served as the source for the data on HBV are the Dessie Referral Hospital, and Kemise General Hospital. During the survey from September 2020 to February 2021, all adults aged 18 and older years that were tested for HBV at these two Hospitals were included in the study. The collection of information in this study was based on secondary data from the two hospitals. The collection of a secondary form of data involved a review of official files which includes laboratory results, patient files, patient diagnostic papers, and different preceding data collecting.

### 2.2 Study design and sample size

This study was designed as a retrospective cross‐sectional Hospital‐based study among adult patients. The sample size was calculated using a two‐independent population proportion. We have two populations from which dichotomous (binary) responses will be recorded. The probability (or risk) of obtaining the event of interest in Population 1 (Dessie Referral Hospital) is P_1_, and in Population 2 (Kemise General Hospital) is P_2_. The corresponding failure proportions are given by *q*_1_ = 1 − *P*_1_ and *q*_2_ = 1 − *P*_2_. Random samples of *n*_1_ and *n*_2_ individuals are obtained from these two populations.

The study population was all adults who were attending the Dessie Referral Hospital and Kemise General Hospital, during the study period. The sample size was calculated using a single proportion population formula based on the following assumptions: the assumption *Z* distribution with a 95% confidence interval (CI) was 1.96%, the margin of error (*d*) was 2%, and proportion (*p*) has taken 50% to maximize the sample size. The final sample size was computed using the formula $n=\frac{Z_{\alpha /2}^{2}P(1-P)}{d^{2}}$. After including 5% of the nonresponse rate, the final minimum sample size was 576 + 504 = 1080.

A simple random sampling technique was used to select an appropriate sample.

### 2.3 Inclusion criteria

✓ Individuals aged≥18 were included.
✓ Individuals who had tested for HBV during the study period.

### 2.4 Exclusion criteria

✓ Individuals who aren't tested for HBV.
✓ Individuals aged <18 were excluded
✓ Individuals who were incomplete measurements.

### 2.5 Variables in the study

Variables that are taken into consideration in this study were decided on based on literature that has been conducted at the local level and worldwide.

#### 2.5.1 Dependent variable

The response variable of this study is the status of adults with HBV for the adult age ≥18 is represented by a random variable $Y_{i}$ with two possible values coded as 1 and 0. So, the response variable of the i*t*h adult $Y_{i}$ was measured as a dichotomous variable with possible values $Y_{i}=1,$ if an *i*th adult had HBV and $Y_{i}=0$ otherwise.

#### 2.5.2 Explanatory variables

The predictor variables are variables that are presumed to affect or determine a dependent variable.

✓ Demographic and sociological characteristics: age, gender, educational status, marital status, religion, current residence, type of hospital, and family history of HBV.
✓ Risk behaviors and history of medical examination and treatment: history of hospitalization, HIV status, hypertension status, diabetes status, TB status, health insurance, and jaundice status.

### 2.6 Statistical models

#### 2.6.1 Binary logistic regression model

Logistic regression analysis extends the techniques of multiple regression analysis in which the outcome variable is categorical. Logistic regression allows one to predict a discrete outcome, such as group membership, from a set of predictor variables that may be continuous, discrete, dichotomous, or a mix of any of these. Generally, when the dependent variable is dichotomous (such as yes/no, presence or absence, success or failure, etc.) binary logistic regression is used.^10^

#### 2.6.2 Parameter estimation in the logistic regression model

The maximum likelihood and non‐iterative weighted least squares are the two most computing estimation methods used in fitting the logistic regression model. The maximum likelihood estimation method is appropriate for estimating logistic model parameters due to the less restrictive nature of the underlying assumptions.^10^

#### 2.6.3 Odds ratios

The odds ratio is the ratio of the odds of an event occurring in one group to the odds of occurring in another group. The odds ratio (OR) is a popular measure of the strength of association between exposure and disease.

In binary logistic regression, the OR is the exponential of the estimated coefficient $\hat{\beta }(\text{exp}(\hat{\beta }))$. An OR of one corresponds to an explanatory variable that does not affect the outcome variable.

#### 2.6.4 Test overall model fit

The goodness of fit or calibration of a model measures how well the model describes the response variable. In testing the hypothesis that the model fits the data, the two common approaches are the likelihood‐ratio statistic ($G^{2}$) and Pearson's and deviance chi‐square statistics ($\chi^{2}$) which are based on the comparison of the fitted and the observed counts.

#### 2.6.5 Likelihood‐ratio test

The most common assessment of overall model fit in multinomial logistic regression is the likelihood ratio test, which is simply the *χ* ^2^ difference between the null model (i.e., with the constant only) and the model containing the predictors (full model).

$G^{2}-2Log\left(\frac{L0}{L1}\right)=-2\left[Log(L0)-Log(L1)\right]=-2(L0-L1)$. Where *L*_0_ is the likelihood of the null model and *L*_1_ is the likelihood of the full model.

#### 2.6.6 The Wald test

Wald statistic is an alternative test that is commonly used to test the significance of individual logistic regression coefficients for each predictor. A Wald test is used to test the statistical significance of each coefficient (β) in the model. The Statistic is defined as $W={\left[\frac{{\hat{\beta }}_{j}}{S.e\;\left({\hat{\beta }}_{j}\right)}\right]}^{2}$, j=1,2,…,p. where βj is the estimated coefficient for the first variable and $S.e\;\left({\hat{\beta }}_{j}\right)$ is its standard error.

### 2.7 Statistical data analysis

The data were checked, cleaned, coded, entered, and analyzed by using SPSS version 20. Descriptive statistics such as frequencies and percentages for discrete data were calculated. Bivariate logistic regression was performed to identify potential candidate variables and each variable with a *p* value less than 0.05 was interred into a multivariable logistic regression analysis to determine the factors significantly associated with the hepatitis B virus. Finally, variables with *p* values < 0.05 in the multivariable logistic regression model were taken as statistically significant.^10^

## 3 RESULTS AND DISCUSSIONS

### 3.1 Descriptive statistics

A total of 1283 adults had been admitted and tested for HBV from the two hospitals. Out of which, 1080 adults have complete measurements and had been taken into consideration on this examination, and others had been excluded from the study because of exclusion criteria.

**Figure 1:**
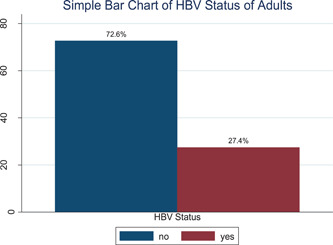
Simple bar graph for hepatitis B virus (HBV) patient status of adults.

Based on Figure 1 results show that the prevalence of HBV in this study is 27.4%.

Table 1 shows the major socioeconomic and demographic factors of the adults. Among 1080 adults 631 (58.4%) were female and 449 (41.6%) were male. Male was found to have a greater prevalence of HBV (19.2%) than female adults (8.2%).

**Table 1:** Distribution of socioeconomic and demographic related determinant factors of HBV

Variables	Categories	Counts (%)	Being experienced HBV		*df*	*χ* ^2^	*p* value
			Yes (%)	No (%)			
Age	18–24	275 (25.5)	3.3	22.1	3	60.166	0.000*
	25–34	374 (34.6)	8.5	26.1			
	35–44	189 (17.5%)	7.3	10.2			
	≥45	242 (22.4)	8.2	14.2			
Gender	Male	449 (41.6)	19.2	22.4	1	135.000^a^	0.000*
	Female	631 (58.4)	8.2	50.2			
Hospital type	Referral	504 (46.7)	11.6	35.1	1	3.225^a^	0.073
	General	576 (53.3)	15.8	37.5			
Educational status	Illiterate	517 (47.9)	14.8	33.1	3	13.595^a^	0.004*
	Primary	241 (22.3)	6.4	15.9			
	Secondary	266 (24.6)	4.6	20.0			
	Higher education	56 (5.2)	1.6	3.6			
Religion	Muslim	787 (72.9)	21.5	51.4	2	10.202^a^	0.006*
	Orthodox	218 (20.2)	3.8	16.4			
	Others	75 (6.9)	2.1	4.8			
Current residence	Rural	684 (63.3)	18.1	45.2	1	1.4593^a^	0.227
	Urban	396 (36.7)	9.3	27.4			
Marital status	Single	173 (16.0)	3.0	13.1	3	13.1054^a^	0.004*
	Married	779 (72.1)	21.9	50.2			
	Separated/divorced	93 (8.6)	1.8	6.9			
	Widowed	35 (3.2)	0.7	2.5			

Abbreviations: *df*, degree of freedom; HBV, hepatitis B virus.

* Significant at 5%.

The mean age of the study adults was 34.24 (SD ±12.56) years which were between 18 and 68 years. Of this 374 (34.6%) were aged between 25 and 34, 275 (25.5%) were aged between 18 and 24, and the rest 242 (22.4%), 189 (17.5%) were aged≥45, and aged between 35 and 44, respectively. And the prevalence of HBV was high among adults of age between 25 and 34 and aged≥45 was 8.5% and 8.2% and followed by age between 35 and 44 with a 7.3% prevalence of HBV.

Of total adults, 684 (63.3%) live in a rural area, while 396 (36.7%) live in an urban area. People living in rural areas had a greater prevalence of HBV (18.1%) than adults living in urban areas (9.3%).

Of total adults, 504 (46.7%) were from Dessie referrals and 576 (53.3%) from Kemise general hospitals with the prevalence of HBV being 15.8% and 11.6%, respectively.

Table 1 additionally shows, that among the 1080 adults 72.9% of them were Muslims, and a higher prevalence of HBV was observed (21.5%), and among the 1080 adults72.1% of them had been married, and 16.0%, 8.6%, and 3.2% had been single, separated/divorced, and widowed, respectively. The prevalence was excessive at (21.9%) for married participants.

Furthermore, Table 1 suggests that the proportions of adults who suffered from HBV are varied by educational status. Majority of respondents 47.9% of them had no education. While only 24.6, 22.3%, and 5.2% of them had secondary, primary, and higher education levels, respectively. The maximum prevalence was determined for adults who had illiterate (14.8%).

Among the socioeconomic and demographic determinant factors gender, age, educational status, marital status, hospital type, and religion were found to have a significant effect on the incidence of HBV at a 5% level of significance.

Table 2 showed that out of the 1080 adults 126 (11.67%) were diabetes positive and out of this 39 (3.6%) were HBV and diabetes, coinfected patients. Among all adults 55 (5.1%) were HIV‐positive of these 15 (1.4%) were HBV‐HIV coinfected adults and also of the total respondents, 113 (10.5%) were had hypertension of these 39(3.6%) were HBV‐hypertension coinfected. Similarly, from the total adults 45 (4.2%), 59 (5.5%) were TB and jaundice status positive of these 15 (1.4%), 37 (3.4%) were HBV‐TB and HBV‐jaundice coinfected patients, respectively.

**Table 2:** Distribution of environmental and health‐related risk factors of HBV in northeastern Ethiopia

Variables	Categories	Counts (%)	Being experienced HBV		*df*	*χ* ^2^	*p* value
			Yes (%)	No (%)			
Health assurance	Yes	605 (56.0)	13.7	42.3	1	5.9948^a^	0.014*
	No	475 (44.0)	13.7	30.3			
Family history of HBV	Yes	142 (13.1)	6.4	6.8	2	69.8220^a^	0.000*
	No	587 (54.4)	16.7	37.7			
	Unknown	351 (32.5)	4.4	28.1			
History of hospitalization	Yes	269 (24.9)	7.5	17.4	2	4.8105^a^	0.090
	No	442 (40.9)	11.9	29.0			
	Unknown	369 (34.2)	8.0	26.2			
Diabetes status	Yes	126 (11.7)	3.6	8.1	2	16.9059^a^	0.000*
	No	815 (75.5)	22.1	53.3			
	Unknown	139 (12.9)	1.7	11.2			
HIV status	Yes	55 (5.1)	1.4	3.7	2	2.2797	0.320
	No	738 (68.3)	19.6	48.7			
	Unknown	287 (26.6)	6.4	20.0			
Hypertension status	Yes	113 (10.5)	3.6	6.9	2	3.3970	0.183
	No	901 (83.4)	23.3	61.1			
	Unknown	66 (6.1)	1.5	4.6			
TB status	Yes	45 (4.2)	1.4	2.8	2	2.6213	0.270
	No	941 (87.1)	24.2	63.0			
	Unknown	94 (8.7)	1.9	6.9			
Jaundice status	Yes	59 (5.5)	3.4	2.0	2	145.0203^a^	0.000*
	No	607 (56.2)	20.9	35.3			
	Unknown	414 (38.3)	3.1	35.3			

Abbreviations: *df*, degree of freedom; HBV, hepatitis B virus.

* Significant at 5%.

The proportion of adults who suffered from HBV varies for adults which have family records of HBV, don't have family records of HBV, and are unknown about their family records of HBV. The highest prevalence of HBV was found at (16.7%) in patients who don't have their family found HBV, (6.4%) who have their family found HBV, and (4.4%) in patients who have unknown family records.

There is a significant association between the incidence of HBV and health assurance (*p* < 0.05). Among 1080 patients, about 56% of them are health assurance and the same prevalence was found (13.7%) in adults who were no health assurance and health assurance.

Moreover, Table 2 showed that the highest proportion of adults who suffered from HBV was observed among no jaundice which means (20.9%) followed by jaundice patients (3.4%) as opposed to the lowest proportion which was recorded in adults who have unknown jaundice status (3.1%).

Of the total patients (75.5%) have no diabetes and only 11.7% have diabetes, the rest have unknown about their diabetic status. Similarly, the highest prevalence of HBV was observed among adult patients who do not have diabetes (22.1%) and (3.6%) have diabetes as compared with adults who had unknown about their diabetes status (1.7%).

From the above Tables 1 and 2 showed that socioeconomic and demographic predictor variables like age, gender, marital status, educational status, religion, hospital type, and risk behaviors predictors like health assurance, history of hospitalization, family history of hospitalization, diabetes, Jaundice were associated with the HBV at a 5% level of significance and considered for multiple logistic regression analysis.

Shows gender was found to be significantly associated with the likelihood of having HBV in the logistic model. Male adults were 4.357 times more likely than female adults to have HBV, even after correcting for other covariates in the model (OR = 4.357; 95% CI: 3.035–6.254).

Adult aged between 25 and 34 was 3.604 times more likely than age between 18 and 24 (OR = 3.604; 95% CI: 1.487–8.736) and adults aged between 35 and 44 were 6.672 times more likely than age between 18 and 24 (OR = 6.672; 95% CI: 2.655–16.763). Similarly, adults aged≥45 were 3.857 times more likely exposed to HBV than adults aged between 18 and 24 (OR = 3.857; 95% CI: 1.509–9.855) controlling for other covariates in the model.

Table 3 also shows that the family history of HBV has a significant association with the incidence of HBV. Adults who had a family history of HBV were 1.965 times more likely to have HBV than adults who were no family history of HBV controlling for other variables in the model (OR = 1.965; 95% CI: 1.230–3.140). Similarly, an adult who was an unknown family history of HBV was 56.8% less likely to have HBV than adults who were no family history of HBV controlling for other variables in the model (OR = 0.432; 95% CI: 0.286–0.653).

**Table 3:** Maximum likelihood estimates of predicting the incidence of HBV

Variables	Categories	*β* (*SE*)	Wald	*df*	Sig.	EXP(β)	95% CI for EXP(*β*)	
							Lower	Upper
Age	18–24 (ref)			3				
	25–34	1.282 (0.452)	2.84		0.005*	3.604	1.487	8.736
	35–44	1.898 (0.470)	4.04		0.000*	6.672	2.655	16.763
	≥45	1.350 (0.479)	2.82		0.005*	3.857	1.509	9.855
Gender	Female(ref)							
	Male	1.472 (0.184)	7.98	1	0.000*	4.357	3.035	6.254
Marital status	Single (ref)			3				
	Married	−0.835 (0.483)	−1.73	1	0.084	0.434	0.168	1.119
	Separated/divorced	−1.466 (0.569)	−2.58	1	0.010*	0.231	0.076	0.704
	Widowed	−0.707 (0.689)	−1.03	1	0.815	0.493	0.128	1.903
Educational status	Illiterate (ref)			3				
	Primary	0.062 (0.224)	0.28	1	0.782	1.064	0.685	1.652
	Secondary	−0.182 (0.249)	−0.73	1	0.454	0.833	0.511	1.357
	Higher education	0.037 (0.386)	0.10	1	0.924	1.037	0.487	2.210
Religion	Others(ref)			2				
	Muslim	−0.337 (0.328)	−1.03	1	0.303	0.713	0.375	1.356
	Orthodox	−1.011 (0.369)	−2.74	1	0.124	0.364	0.176	1.035
History of hospitalization	No (ref)			2				
	Yes	−0.439 (0.220)	−1.99	1	0.045*	0.644	0.418	0.993
	Unknown	−0.596 (0.200)	−2.98	1	0.003*	0.551	0.372	0.816
Current residence	Urban (ref)							
	Rural	0.007 (0.187)	0.04	1	0.970	1.007	0.698	1.452
Family history of HBV	No (ref)			2				
	Yes	0.676 (0.239)	2.83	1	0.005*	1.965	1.230	3.140
	Unknown	−0.839 (0.211)	−3.97	1	0.000*	0.432	0.286	0.653
Health assurance	No (ref)							
	Yes	−0.570 (0.173)	−3.29	1	0.001*	0.565	0.403	0.794
Diabetes	No (ref)			2				
	Yes	−0.127 (0.251)	−0.51	1	0.611	0.880	0.538	1.439
	Unknown	−0.861 (0.312)	−2.76	1	0.006*	0.423	0.229	0.779
Hypertension status	No (ref)			2				
	Yes	0.261 (0.264)	0.99	1	0.323	1.298	0.774	2.176
	Unknown	−0.031 (0.352)	−0.09	1	0.931	0.970	0.487	1.932
Jaundice (liver problem)	No (ref)			2				
	Yes	0.916 (0.329)	2.82	1	0.005*	2.500	1.312	4.765
	Unknown	−1.786 (0.222)	−8.04	1	0.000*	0.168	0.108	0.259
Constant	–	−0.979 (0.483)	−2.03	1	0.043*	0.376	0.079	0.599

Abbreviations: CI, confidence interval; *df*, degree of freedom; HBV, hepatitis B virus; *SE*, standard error.

* Significant at 5%.

The logistic model showed that adults who have Health assurance are negatively associated with the incidence of HBV. An adult having a health assurance was 43.5% less likely to have HBV than adults who had no Health assurance (OR = 0.565; 95% CI: 0.403–0.794).

Similarly, the logistic model has shown that adult history of Hospitalization is also negatively associated with the occurrence of HBV. An adult having a history of Hospitalization was 35.6% less likely to have HBV than adults who had no history of Hospitalization (OR = 0.644; 95% CI: 0.418–0.993) on the same way, those who had an unknown history of Hospitalization were 44.9% less likely to have HBV than adults who had no history of Hospitalization (OR = 0.551; 95% CI: 0.372–0.816).

The logistic model showed that Jaundice status is a significant predictor of the incidence of HBV. An adult who is Jaundice was 2.500 times more likely to have experienced HBV than an adult who had no Jaundice controlling for other variables in the model (OR = 2.500; 95% CI: 1.312–4.765). Similarly, adults having unknown about their jaundice status were 83.2% less likely to suffer HBV than adults who had no control for other variables in the model (OR = 0.168; 95% CI: 0.108–0.259).

## 4 DISCUSSIONS

Hepatitis B virus infection is highly endemic in Northeastern Ethiopia, nearly 3 in 10 adults (27.4%) were positive with HBs Ag. In North‐central, Nigeria seropositive was 18.4%,^11^ in Northern Uganda prevalence of HBs is 17.6%,^12^ in Cameroon overall prevalence was 12.6%.^13^ But it is lower than the studies done in Northeast China among an Adult Population where the prevalence of the HBs was 35.66%.^14^ This much difference may be due to the study target group and the people living standards.

The prevalence of HBsAg observed in the current study is generally categorized as high endemic according to the WHO criteria (≥8.0%).^7^ This may be due to the difference in the behavioral and cultural practices in the country.

This study found that experiencing HBV was significantly associated with age. The log‐likelihood of HBV among adults aged between 25 and 34 was 3.60 times more likely than the younger (18–24) (OR = 3.60, 95% CI: 1.487–8.736) and among those aged between 35 and 44, the log‐likelihood of having HBV was 6.67 times more likely than the reference category (OR = 6.67, 95% CI: 2.65–16.76). Similarly, adults aged≥45 were 3.56 times more likely to have HBV than those aged 18–24 (OR = 3.85, 95% CI: 1.51–9.85). This result is in agreement with,^15^, ^16^, ^17^ and also a population‐based study done in Brazil show the older age group 60–69 were more likely to have HBV than younger age groups,^18^ which revealed that older age has one of the factors leading to increased risks of HBV, but inconsistent with the study in the northeast, China.^14^, ^17^ Older carriers are more likely than younger carriers to clear HBeAg. This may the difference in the behavioral and cultural practices in the country.

The prevalence of HBV was high among male adults 4.36 times more likely than females this result is in agreement with.^14^, ^17^, ^18^ This study is also comparable with studies conducted in Gondar teaching Hospital, Ethiopia, and Pasteur institute, Morocco, respectively.^19^, ^20^ This may result in males traveling more frequently than females in developing countries, especially in rural and semi‐urban communities, due to their job nature. The second significant reason is that men frequently engage in high‐risk behaviors for HBV transmission (smoking, alcohol use, and chat chowing), as well as a tendency to seek medical help when they are in poor health.

Similarly, adults who had a family history of HBV were 2.025 times more likely to suffer from HBV than adults who don't have a family history of HBV (*p* = 0.003). This is in agreement with the study of other parts of Ethiopia,^21^ in Asia Vietnam,^17^ in Northeast China,^14^ and in Africa Uganda.^22^ In contrast, this is inconsistent with other studies.^23^, ^24^ This could be due to a lack of understanding about HBV transmission methods, a lack of caution when sharing sharp objects, traditional practices, or unsafe sexual practices. In Ethiopia, it is believed that hepatitis is not spread from person to person, but rather that it is Bat's disease, or “Yewef Beshita” in Amharic.^25^

In the current study, history of hospitalization was negatively associated with the prevalence of HBV. This finding goes in line with the study Arba Minch Hospital,^24^ in the study Gedeo Zone Southern Ethiopia,^26^ but contradicted a study conducted at Deer Hospital Eastern Ethiopia,^27^ studies at Wolaita Zone Public Hospitals, Southern Ethiopia.^28^ This is an indication of a difference among hospitals that needs intervention.

In this study, Jaundice was positively associated with the incidence of HBV and this is comparable with the study done as opposed to the study in other Southern parts of Ethiopia,^15^ and Northeast China.^14^ Additionally, the study conducted in Northwest Ethiopia showed that study subjects who had a previous history of jaundice were five times more likely to have HBV infection compared to those who had not.^29^

We assessed that the prevalence of HBV did not vary significantly in socio‐demographic and risk behavior variables including marital status, educational status, current residence, religion, HIV status, Hypertension status, and, TB status. The prevalence of HBV infection was found not to be different between rural and urban areas. This finding was consistent with the study in Northeast China.^14^ Some studies have reported that there was a significant seropositivity difference between rural and urban regions.

This study showed that education was not an independent influencing factor on Hepatitis B virus this is in line with the study done in China,^30^ in Bahrain,^31^ and opposed to the study done in Cameron,^32^ in Nigeria, in Jimma Ethiopia,^33^ and Southern Ethiopia.^15^

## 5 CONCLUSIONS

The study identified that demographic, environmental, and health‐related variables have an important effect on determinants of HBV in Northeastern Ethiopia. According to this study, age, gender, family history of HBV, History of Hospitalization, health assurance, and Jaundice were all important factors in determining the incidence of HBV in Northeastern Ethiopia. While, educational status, marital status, current residence, hypertension status, religion, diabetes status, HIV status, and TB status were found to be insignificant factors in determining HBV in Northeastern Ethiopia.

## AUTHOR CONTRIBUTIONS

**Hussein Mohammed**: Conceptualization; formal analysis; investigation; methodology; resources; software; validation; visualization. **Aragaw Eshetie**: Formal analysis; methodology; supervision; validation; visualization; writing—review & editing. **Dessie Melese**: Investigation; methodology; supervision; validation; visualization; writing—original draft; writing—review & editing.

## CONFLICTS OF INTEREST

The authors declare no conflicts of interest.

## ETHICAL STATEMENTS

The study was conducted after obtaining institutional ethical clearance from the University of Gondar College of Natural and Computational Science. After Separate permission was also obtained from Northeastern Referral Hospital, and General Hospital information about the patients was a review of official files which includes patient laboratory results, patient files, patient diagnostic papers, and different preceding data collected.

## TRANSPARENCY STATEMENT

The corresponding author affirms that the results in this manuscript are an honest and accurate display of the study being reported. No important aspects of the study have been omitted. Any discrepancies from the study as planned have been explained.

## Data Availability

The data used to support the findings of this study are available from the corresponding author upon request.
